# Juvenile Myoclonic Epilepsy in Rural Western India: Not Yet a Benign Syndrome

**DOI:** 10.1155/2016/1435150

**Published:** 2016-10-13

**Authors:** Devangi Desai, Soaham Desai, Trilok Jani

**Affiliations:** ^1^Department of Medicine, Pramukhswami Medical College, Shree Krishna Hospital, Karamsad, Anand, Gujarat 388325, India; ^2^Department of Neurology, Pramukhswami Medical College, Shree Krishna Hospital, Karamsad, Anand, Gujarat 388325, India

## Abstract

*Purpose*. To study prevalence of uncontrolled seizures in patients with juvenile myoclonic epilepsy [JME] and assess factors responsible for it.* Methods*. An ambispective study of all patients with JME attending our epilepsy clinic was done. We recruited all patients with JME evaluated between 1 January 2009 and 31 December 2013 and followed them up to 31 December 2015.* Results*. Amongst 876 patients with epilepsy, JME was present in 73 patients. Amongst them, 53 [72.6%] had uncontrolled seizures prior to neurology consultation. Factors responsible for uncontrolled seizures included pitfalls in diagnosis like absence of prior neurology consultation missed history of myoclonus in prior consults and pitfalls in interpretation of EEG. Pitfalls in management were incorrect antiepileptic drug use, underdosing of AED, noncompliance with lifestyle, noncompliance with medicines, associated psychogenic nonepileptiform events, patients deliberately missing medicines for secondary gain, and concomitant alternative medicine use. 45 (84.9%) patients had “pseudorefractoriness.” True refractoriness [seizures despite 2 correctly dosed rational drugs] was seen in 8 (15.1%) patients only.* Conclusion*. Three-fourth of our patients had uncontrolled seizures initially, predominantly due to pitfalls in its diagnosis and management. Improving patient awareness and primary physician training for JME management is the need of the hour.

## 1. Introduction

Juvenile myoclonic epilepsy (JME) represents the most common idiopathic epilepsy syndrome [1]. JME has been the subject of intensive research over the past 25 years. It is an archetypical epileptic syndrome, with a fairly homogenous presentation and a still largely unknown etiology [2, 3]. Its clinical spectrum now includes cognitive and psychiatric symptoms as significant comorbidities, and the elucidation of its probably multiple genetic mechanisms is an ongoing process [4, 5]. JME is often described as “benign” epilepsy, as seizures in most patients can be managed adequately and patients will not suffer severe limitations in their lifetime expectations [2, 3]. Some clinicians have refrained from calling this disease “benign” due to the need for often lifelong duration of treatment [4].

Clear consensus criteria have been classified for diagnosis and management of JME [1, 4]. As compared to focal epilepsies, patients with JME are described to have uncontrolled seizures if they continue to have >1 generalized tonic clonic seizure (GTCS) per year and/or >1 myoclonic jerk per month after starting of treatment with antiepileptic drugs [4, 6]. True pharmacoresistant forms [meaning persistent seizures despite adequate dose of appropriate antiepileptic medicine for adequate period] account only for 10–15% of all JME cases who have uncontrolled seizures [3]. However, it is quite possible that this number is higher if studied over longer period of follow-up [5, 6]. Studies on seizure outcomes in JME have differed in terms of outcomes assessed. While some authors have assessed long-term seizure remission, some have studied pharmacoresistance in the short term of 1-2 years' period. Pitfalls in diagnosis and treatment are often the reason behind the uncontrolled seizures in these patients, especially in developing countries [7–9]. Problems in diagnosis can be a result of missing the history of myoclonus in evaluation, misinterpretation of Electroencephalography [EEG] findings, delay in diagnosis, and inappropriate consultation [7, 8]. Problems in treatment can be due to inappropriate drug therapy, noncompliance with therapy, and lifestyle adjustment or other factors [8–10]. We aimed to study the frequency of uncontrolled seizures amongst patients with JME in a rural based Epilepsy Clinic, assess factors responsible for the lack of control, and assess the improvement in seizure control with simple methods like rational therapy, education about disease, lifestyle management, drug compliance, and follow-up.

## 2. Methods

Our hospital is a rural based medical teaching hospital attached to Pramukhswami Medical College, in Karamsad village, in Anand district of Gujarat state of Western India serving a predominantly rural-semiurban population of about twenty lacs. The hospital has 550 inpatient beds and receives approximately 900 outpatients daily across all medical specialties. Amongst these patients, neurology department sees approximately 60 outpatients daily. An Epilepsy Clinic is run by the neurology department twice a month on every first and third Tuesday each month where all patients with epilepsy are serially followed up, counseled, and treated. All patients seen in the Epilepsy Clinic are classified on the basis of ILAE classification and followed up serially subsequently. In this ambispective study, we identified and included patients with JME evaluated between 1 January 2009 and 31 December 2013 and followed them up to 31 December 2015 to look for seizure control. Using a predefined structured questionnaire, 2 of the investigators [DD and TJ] individually identified all patients with JME under follow-up in the Epilepsy Clinic after review of demographic data collected from the electronically captured data in the hospital database and EEG software database as well as the patient case record forms to take care that none of the patients with JME on regular follow-up was missed. JME was diagnosed on the basis of JME consensus definition clinical criteria class II [4]. These criteria have the following 5 points: (1) myoclonic jerks occurring predominantly on awakening, (2) myoclonic jerks facilitated by sleep deprivation and stress and provoked by visual stimuli and praxis or GTCSs preceded by myoclonic jerks, (3) EEG showing normal background and at least once interictal generalized polyspike and wave discharges with or without myoclonic jerks, (4) no mental retardation or deterioration, and (5) age at onset between 6 and 25 years. All patients who did not satisfy the diagnostic criteria of JME or those who had not been on regular follow-up at least 3 times a year in between 2009 and 2013 were not included in the study. The patients with age < 6 years or history suggestive of cognitive retardation or abnormal background on EEG were not included in the study. All patients were being followed up by single investigator [SD] in the Epilepsy Clinic. Once these patients were identified, we used a structured questionnaire, to collect relevant details for this study from case records at the time of patient consultations during the serial follow-ups. The outpatient records of these patients were coded so as not to miss any of the patients during the subsequent visits. These patients were followed up serially and details of 2-year seizure control of all patients were collected in the follow-up visits using a structured questionnaire. Seizures were considered as “uncontrolled” in this study, if there were more than one generalized tonic clonic seizure per year and/or more than one myoclonic jerk per month [4, 6]. Psychogenic Nonepileptiform Seizure-Like Symptoms [PNES] were diagnosed on the basis of clinical semiology and findings on video EEG recordings.

**Figure 1:**
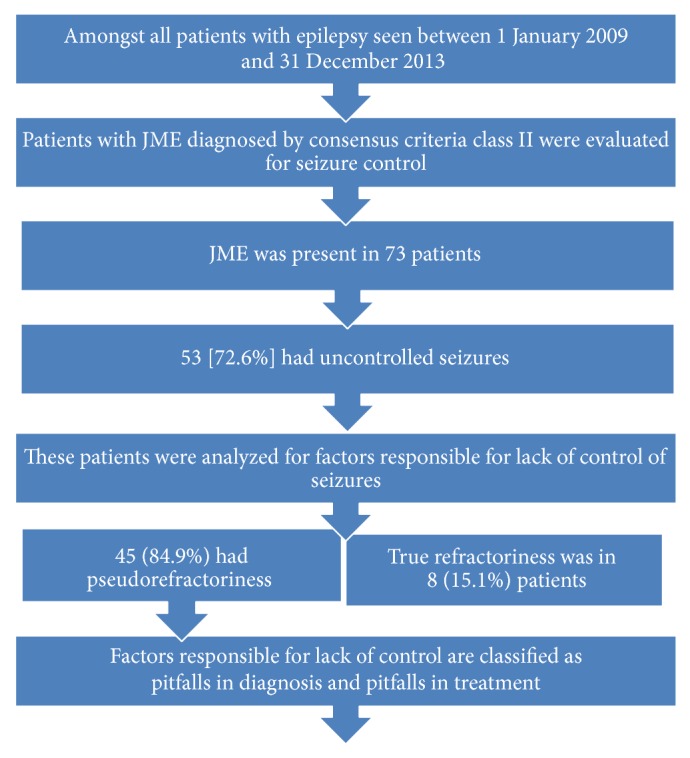
Flowchart detailing the process of evaluation during this study.

All patients and data were evaluated using a structured proforma to assess for the presence or absence of various pitfalls in diagnosis and treatment of JME leading to uncontrolled seizures. The factors responsible for uncontrolled seizures were recorded in a case record form and classified as pitfalls in diagnosis or pitfalls in treatment [11]. Quantification of prevalence of uncontrolled seizures in patients with JME and the factors responsible for uncontrolled seizures in this study population was done during the serial follow-up visits in the Epilepsy Clinic using a structured questionnaire. [See Figure 1.]

## 3. Results

Amongst 876 patients with epilepsy, JME was present in 73 patients [8.3%] [33 males, 40 females] [mean age 27] (range 6–57). Amongst patients with JME, 53 [72.6%] had uncontrolled seizures [>1 generalized tonic clonic seizure/year or >1 myoclonic jerk/month] prior to neurology consultation at our center. Factors responsible for uncontrolled seizures were analyzed. [See flowchart in Figure 1.] 45 (84.9%) patients had “pseudorefractoriness” [seizures were completely controlled after neurology consultation, counseling, rational medication, and lifestyle modification]. True refractoriness [seizures despite 2 correctly dosed rational drugs] was seen in 8 (15.1%) patients only.

Factors responsible for “pseudorefractoriness” were classified into pitfalls in diagnosis and pitfalls in treatment [11]. [See Table 1.] Important pitfalls in diagnosis were absence of a prior neurology consultation and missing of history of myoclonus in prior consults. [See Table 1.] Only 6 (11.3%) patients were diagnosed to have JME prior to consultation in our Epilepsy Clinic. There was a mean delay of 8 years from onset of seizures to diagnosis at our center. We found that the diagnosis of JME was missed in our patients despite the fact that 75% of our patients had undergone an EEG at another hospital prior to consultation here. The misdiagnosis was due to 3 main reasons: a prior EEG was either normal [in 30.1%] or revealed focal abnormality [in 28.3%] or a sleep deprived EEG was not done at prior consultation [in 95%]. When a sleep deprived EEG was done at our center, we could subsequently record generalized polyspike and wave discharges in all these patients.

Important pitfalls in management consisted of use of incorrect antiepileptic drug [in 37 (69.8%)] [phenytoin (11), phenobarbitone (9), carbamazepine (9), and oxcarbazepine (8)] and underdosing of antiepileptic drugs [AED] [in 21 (39.6%)] (lower than recommended dose of AED as per body weight). Besides these, important pitfalls related to lack of information of the disease and required lifestyle modification. 16.9% of patients with JME actually had controlled JME but had comorbid PNES leading to a diagnosis of uncontrolled seizures. 15.1% of our patients were deliberately not taking AEDs intermittently in order to have a seizure/myoclonus.

Once we evaluated these patients, we provided them with rational AEDs, in recommended appropriate doses and counseled them about appropriate lifestyle modifications. We found that in the subsequent 2 years of follow-up, 84.9% of these patients had complete control of seizures. 8 (15.1%) of patients had control of seizures only with education about lifestyle modification as well as drug compliance. Only 8 of the patients continued to have persistent seizures despite being on 2 rational AEDs for more than 2 years which is consistent with the natural history of JME.

## 4. Discussion

This study aimed at assessing the prevalence of uncontrolled seizures in patients with JME diagnosed in a rural based Epilepsy Clinic in Western India found that about three-fourth [72.6%] of patients with JME had uncontrolled seizures prior to consultation here. Camfield and colleagues have reported that 88% of patients had very well controlled seizure over a 25-year period [5, 6]. Similar studies from western literature have documented very well controlled seizures in patients with JME [3, 4, 12]. A study from India also confirms the finding of excellent control of seizures in patients with JME [9]. On the basis of such findings, JME is often described as a “benign” type of epilepsy and often believed not to affect the quality of life of patients in a major way [4, 6]. However, most of these studies are from tertiary care urban epilepsy centers. Our study, in a rural based secondary care Epilepsy Clinic, finds that patients in developing countries, especially in rural areas, have significant morbidity due to lack of control of seizures. Our findings are similar to findings from studies from the western region published in the early 1990s [7, 8, 10]. These findings reveal severe treatment gap in patients with epilepsy in developing countries, especially rural areas which seem to be two decades behind the developed countries in treatment of JME.

We assessed for the important factors responsible for lack of control in these patients. We classified the causes of lack of control into pitfalls in diagnosis and pitfalls in management. We found that only 11% patients had a diagnosis of JME prior to consultation here. Amongst the patients seen, there was a mean delay of 8 years from onset of seizures to diagnosis of JME which was done when seen at our center. Similar findings of lack of initial diagnosis as well as significant delay in diagnosis have also been found in other studies from other parts of India [9, 12]. More than 80% of our patients did not have a neurology consultation prior to visit here. They were being managed by an internal medicine physician, psychiatrist, or an alternative medicine practitioner prior to consultation here.

An important clinical feature of JME which is also important for diagnosis is the elicitation of history of myoclonus seen especially in the morning on awakening early or in sleep deprived state. In three-fourth of our patients, a history of myoclonic jerk was missed to be documented in prior consultation. Elicitation of history of myoclonic jerks in a routine evaluation of patients presenting with seizures is an important issue and is often missed by inexperienced physicians. This problem needs to be addressed in the medical teaching curriculum at graduate level itself so that the diagnosis of JME is not missed.

We found that the diagnosis of JME was missed in our patients despite the fact that three-fourth of our patients had undergone an EEG at another hospital prior to consultation. Focal clinical and EEG abnormalities are not uncommon in JME as found in a study from India and this can be a major reason for misdiagnosis in JME [13]. If the treating physician is not well-experienced in epilepsy care and not aware of the fact that focal findings can occur in JME, such abnormalities would often lead to misdiagnosis of focal epilepsy, leading to use of carbamazepine or oxcarbazepine, which would not be effective in JME, leading to lack of control of seizures [13, 14]. A sleep deprived EEG definitely increases the sensitivity of EEG records, but in private clinical practice patients often undergo the tests immediately after the outpatient consultation and are not called for sleep deprived EEG on another day.

We classified pitfalls in management into physician related factors and patient related factors. Physician related factors included use of inappropriate type of AED (like carbamazepine, oxcarbazepine, phenytoin, and phenobarbitone) and use of inadequate doses of AED [less than recommended dose of AED as per body weight] [7–9, 15]. Nearly seventy percent of our patients did not receive the correct antiepileptic (sodium valproate, levetiracetam, or lamotrigine) which reflects a gap in training of medical persons at the level of general practitioners, physicians, and psychiatrists who are missing the knowledge of rational drug therapy in JME. Amongst patient related factors, noncompliance with medicines and concomitant use of alternative medicines were an important factor of uncontrolled seizures in our study. In other studies also noncompliance with AEDs has been an important factor contributing to lack of seizure control [8]. Various studies have found comorbid psychiatric or personality disorders in patients with JME and this has been suggested to be one of the reasons for noncompliance of AEDs [9, 13, 16]. Approximately half of our patients were not aware of the important lifestyle modifications required for JME, mainly taking adequate sleep and avoiding sleep deprivation, leading to breakthrough seizures. The support of having epilepsy nurse, epilepsy counselor, and social worker which the physicians and patients of developed countries or tertiary epilepsy centers have is often not available in developing countries leading to inadequate patient awareness on lifestyle modifications required in JME. Most of the patient related factors can be controlled by increasing the time the physicians spend in consultation with the patients and having printed patient information sheets specific for JME available for patients at the time of consultation. Training and motivation of the EEG technician and local staff nurses for patient and family education about epilepsy may improve epilepsy care in the resource limited setting.

About one-fifth of our patients actually had controlled JME but had comorbid PNES leading to a diagnosis of uncontrolled seizures. We could diagnose these by appropriate semiology features suggestive of PNES and finding the same on a STVEEG with induction by verbal suggestion. We also found that 15.1% of our patients were deliberately not taking AEDs intermittently in order to have a seizure/myoclonus. On detailed interview, we found that having a diagnosis of epilepsy was making them have special privileges in their family and having a control of seizures was leading to adjustment problems akin to “burden of normality” as experienced in patients undergoing epilepsy surgery [17]. This phenomenon may be responsible for both having PNES and deliberating missing AEDs, both leading to lack of control of seizures.

We found that, in the subsequent 2 years of follow- up, more than three-fourth of our study patients had complete control of seizures. In our study, even though the fraction of patients suffering from persisting uncontrolled seizures at inclusion was very large, only about 15% remained refractory to pharmacotherapy at 2 years of follow-up. The findings of our study are consistent with findings of Genton and Gelisse who report that pharmacoresistant forms account for 10–15% of all JME cases [3]. However, it is quite possible that this number is higher if studied over longer period of follow-up. Several long-term follow-up studies during the last decade have found five-year seizure remission in a range from as low as 27% to 68% [12, 18]. Senf and colleagues followed up patients with JME for a mean period of 45 years and found that nearly 60% patients were seizure-free for a period of 5 years [19]. However, there is significant heterogeneity across these studies in terms of study population, control of seizure type studied [namely, myoclonic seizure or generalized tonic clonic seizure], overall duration of follow-up of individual patients, and consideration of seizure remission or seizure control in the individual studies. The present study shows that simple principles of management of epilepsy like a correct diagnosis, appropriate treatment, lifestyle management advice, and follow-up had a remarkable effect on seizure control in our group of patients of JME in rural area of developing country over 2 years of follow-up.

The fact that our study is single centric study may be considered as one of its limitations. However, the fact that all patients were evaluated at a single center by a single neurologist makes our study population a homogenous one. While most studies on JME are from tertiary epilepsy care centers of developed countries, our study describes the clinical situation in a secondary care Epilepsy Clinic in a rural area in a developing country. Our findings demonstrate that the problem of inappropriate follow-up and treatment of JME is huge in developing countries/rural areas and that a very large amount of patients achieve remarkable control if diagnosed, informed, and treated correctly.

## 5. Conclusion

Though JME is considered a “benign” syndrome, nearly three-fourth of patients in developing countries, especially in rural areas, have uncontrolled seizures, predominantly due to preventable but often neglected pitfalls in its diagnosis and management. Also these groups of patients can achieve remarkable seizure control with simple principles of epilepsy management. The situation of epilepsy care in rural Western India is about two decades behind developed countries. Patient awareness and counseling, as well as primary physician training about diagnosis and treatment of JME, are the need of the hour to reduce the epilepsy treatment gap and improve quality of epilepsy care in rural/developing regions.

## Figures and Tables

**Table 1:** Pitfalls in diagnosis and treatment.

	Number [percentage]
*Pitfalls in diagnosis*	
Absence of prior neurology consultation	42 (81.1%)
Missed history of myoclonus in prior consults	39 (73.5%)
Prior EEG showing focal discharges	15 (28.3%)
Prior EEG being normal	16 (30.1%)
Misinterpreted EEG	10 (18.8%)
*Pitfalls in treatment*	
Incorrect antiepileptic (AED) drug use	37 (69.8%)
Underdosing of AED	21 (39.6%)
Noncompliance with lifestyle	24 (45.3%)
Noncompliance with medicines	18 (33.9%)
Associated psychogenic nonepileptiform events	9 (16.9%)
Patients deliberately missing medicines for secondary gain	8 (15.1%)
Concomitant alternative medicine use	18 (33.9%)
